# Evaluation of a New Technique of Gingival Smile Reduction after Hyaluronic Acid Infiltration: A Cohort Study Focusing on Gingival Exposure and Patient-Oriented Outcomes

**DOI:** 10.3390/dj12100329

**Published:** 2024-10-15

**Authors:** Gema Angulo-Manzaneque, María Baus-Domínguez, Gonzalo Ruiz-de-León-Hernández, María-Ángeles Serrera-Figallo, Daniel Torres-Lagares, Fátima S. Aguilera

**Affiliations:** 1 Faculty of Dentistry, University of Seville, C/Avicena s/n, 41009 Seville, Spain; gema.angulomanzaneque@gmail.com (G.A.-M.); maserrera@us.es (M.-Á.S.-F.); danieltl@us.es (D.T.-L.); 2 Faculty of Dentistry, Department of Stomatology, University of Granada, Colegio Máximo de Cartuja s/n, 18071 Granada, Spain; fatimas@ugr.es

**Keywords:** gingival smile, hyaluronic acid, infiltration techniques, myomodulation, injection of facial fillers, patient-centered outcomes

## Abstract

A gummy smile is the visualization of more than 3 mm of gingiva above the maxillary incisors. This study aimed to evaluate the long-term effects on aesthetics and patient satisfaction of a new hyaluronic acid infiltration technique in reducing the gummy smile. Fifty patients with gummy smiles were treated using hyaluronic acid infiltration points by applying 0.1 mL per injection at specific points. Post-treatment evaluations were performed one week, one month, three months, six months, and 12 months later. A questionnaire assessed patient satisfaction, willingness to recommend the treatment, and side effects (pain and bruising during and after treatment). An average reduction of 2.5 mm in gingival exposure during smiling was observed. After one week, the reduction was constant. At six months, 40% of the original gingival exposure was recovered, and recovery was complete at 12 months. Patient satisfaction was 82.1%, and 100% of patients would recommend the treatment. Bruising, swelling, and pain decreased to 0% from the first month. In conclusion, this technique is a safe and effective option to improve the smile’s appearance with minimal complications.

## 1. Introduction

Gummy smile (GS) is a cosmetic condition in 10.5–29% of young adults, with a prevalence of 14% in women and 7% in men [1]. It is characterized by excessive gingival exposure during smiling. This exposure, commonly defined as the visibility of more than 3 mm of gingiva, can profoundly impact the self-esteem and social perception of the affected individuals [2,3]. The prevalence of GS decreases with age due to the lowering of the upper and lower lips, which reduces the exposure of the gingiva and maxillary incisors [4,5]. The gummy smile is a complex phenomenon whose etiology involves multiple anatomical and functional factors, profoundly affecting aesthetics and facial harmony [5,6]. This condition influences a person’s social perception and self-esteem and challenges clinical management.

**Figure 1:**
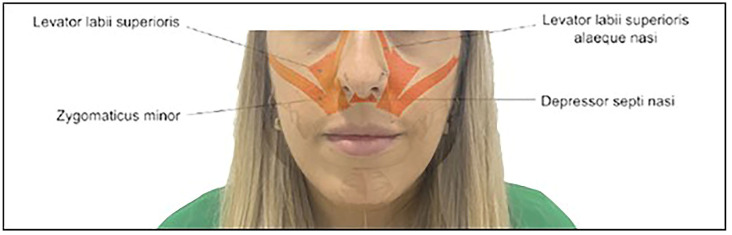
Muscles involved in the gingival smile.

Functionally, the facial muscles, particularly the levator labii superioris and ala nasalis (LLSAN) muscle, play a crucial role. The hyperactivity of these muscles can lead to excessive upper lip elevation, revealing a significant amount of gingiva. The dynamics of these muscles profoundly impact the aesthetics of the smile [7,8]. Previous work has underscored the importance of considering a therapeutic approach that addresses the static and dynamic aspects of the gingival smile [7,8,9,10], addressing the condition’s underlying causes to improve both the function and aesthetic appearance. The cause of a gummy smile may stem from a short lip, a hypermobile lip, or both [11]. Therefore, it becomes imperative to determine the muscles involved in the smile (Figure 1).

Anatomical evaluation shows that the LLSAN is the only muscle that elevates the lateral and upper lip medially, together with the nasalis muscle. Other muscles involved in smile dynamics are the levator labii superioris (LLS), zygomaticus major (Zma), and zygomaticus minor (Zmi), which elevate the lip from the commissures distally [2,3,12,13,14]. The smile is formed in two stages. During the first stage, the contraction of the levator muscles elevates the upper lip to the nasolabial fold. The second stage involves the further superior elevation of the lip and crease by three muscle groups: (1) the upper lip elevator muscles of the upper lip, which originate in the infraorbital region; (2) the zygomaticus major muscles; and (3) the superior fibers of the buccinator [6]. The most common type of gummy smile, the anterior (or central) type, usually involves the nasal septal depressor muscles and the levator labii superioris and ala nasalis levator (LLSAN) muscles.

The treatment of the gummy smile has evolved significantly over the years, with an increasing focus on less invasive and more aesthetic treatments. Traditionally, surgical options such as gingivectomy and orthognathic surgery have been used to address the underlying anatomic causes of the gummy smile. These invasive procedures can involve prolonged recovery and considerable surgical risks [15,16].

In addition, orthodontic tools, such as incisor intrusion and mini-implants, offer effective options for treating gingival smiles by repositioning teeth and reducing gingival display. These techniques have been shown to be a reliable and stable alternative, providing a significant reduction in deep overbite and gingival visibility [17,18].

In recent times, botulinum toxin type A has gained popularity as an effective non-surgical treatment to moderate the muscle activity involved in upper lip elevation. This technique offers a temporary but effective correction with fewer risks and a faster recovery than surgical options [19,20].

On the other hand, hyaluronic acid infiltration, initially proposed by Diaspro et al. (2018) [1], has emerged as another valuable therapeutic option. This technique takes advantage of hyaluronic acid’s volumizing and myomodulating properties to adjust the upper lip dynamics and gingival exposure, providing natural and customized results according to each patient’s specific needs. Hyaluronic acid allows subtle and reversible aesthetic adjustments, unlike more invasive treatments. It is preferred for those seeking aesthetic enhancement without the extended recovery times or risks associated with surgery [21].

This new technique involves injecting hyaluronic acid into the periosteum in the piriform fossa area, compressing the lateral fibers of the levator labii superioris and ala nasalis (LLSAN) [1], inhibiting the mobility of the deep portion of the LLSAN muscle and that of the LLS muscle, and mitigating the elevation of the upper lip during smiling. Together with the injection of the nasal spine into the Septo Nasal depressor muscle, this technique results in an immediate improvement of the gingival smile.

Therefore, considering that the perception of an attractive smile is crucial not only for social interaction, but also for personal well-being, influencing how others perceive individuals and how they perceive themselves [7,8], the objectives of this study were (1) to confirm the feasibility and efficacy of a novel hyaluronic acid infiltration technique in the muscles involved in the gingival smile and (2) to better understand the duration of effects and patient perception of the present noninvasive treatment for the gingival smile. The null hypothesis tested was that there were no differences over time in GS measurements after the hyaluronic acid infiltration treatment of the smile muscles.

## 2. Materials and Methods

### 2.1. Patient Profile and Study Protocol

In this longitudinal study of a case series (prospective cohort study), 50 patients affected by gingival smiles with gingival exposure greater than or equal to 3 mm when smiling were enrolled, taking into account the inclusion and exclusion criteria (Table 1). Patients presenting GS were selected from the Oral Surgery Service of the Faculty of Dentistry of Seville between January 2022 and May 2024. All participants maintained an excellent oral health status, were free of active periodontal diseases, and did not present conditions contraindicated using hyaluronic acid. This work was performed with prior informed consent from the patients detailing the procedures, potential benefits, and risks involved in the study. The study was approved by the CEIm of HM Hospitales (protocol 23.11.2258-GHM).

For inclusion in the study, the patients completed a Gingival Smile Treatment Selection Survey in which they answered seven multiple-choice questions about patient expectations, concerns, and limitations about treatments (surgical or not) for gingival smile and five single-choice (yes/no) questions about preferences for different treatments once each of the treatments had been explained to them: periodontal coronary lengthening; botulinum toxin treatment; orthognathic surgery; lip replacement surgery; and hyaluronic acid infiltration (Table 2).

In addition, another survey on the psychological impact of the gingival smile was conducted. It consisted of 7 questions with multiple-choice answers dealing with self-esteem, social perception, how the patient feels, and whether he/she has sought a solution (Table 3).

Following the completion of the pretreatment surveys, the appropriate clinical and dental history was taken, and a complete facial and dental examination was performed, along with photographs of the patients selected for this noninvasive gummy smile treatment. An assessment of the etiology of the gummy smile and the determination of the muscles involved were also conducted.

### 2.2. Materials and Treatment Protocol

We ensured that all patients fully understood the concept of “reversible procedure” through a detailed description in the informed consent form. This document clearly explained the nature of the treatment, including the reversibility of the effects of hyaluronic acid. In addition, before treatment, we took the time to address any questions patients might have had to ensure that they fully understood the procedure, its effects, and its reversibility.

Apriline^®^ Forte (Sellaesthetic; Valencia, Spain), a single-phase 100% cross-linked hyaluronic acid gel that uses technology to cross-link hyaluronic acid molecules in a single phase effectively, was used. Apriline^®^ Forte has a hyaluronic acid concentration of 23 mg/mL and a molecular weight of 1,000,000 kDa. It was administered using a 27 G-gauge needle. Each patient received a 1.0 mL volume of Apriline^®^ Forte, ensuring product sterility and reducing the risk of infection.

**Figure 2:**
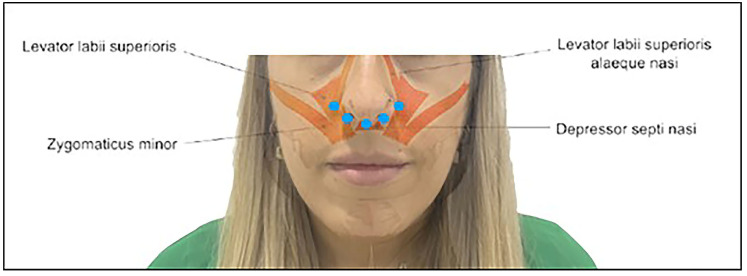
Hyaluronic acid injection sites (blue dots).

The technique used involved infiltrating hyaluronic acid, applying small amounts of 0.1 mL per injection in specific points at the level of the nasal spine, piriform fossa (canine fossa), and base of the nose, corresponding to teeth 13, 12, 11, 21, 22, and 23, for a total of 1 mL per patient. The procedure was performed without anesthesia in the dental office (Figure 2).

The injection sites are described in the following subsections.

#### 2.2.1. Piriformis Fossa or Canine Fossa

Injection into the piriform fossa can decrease LLSAN activity, improve the sunken appearance, and enhance its appearance. The LLSAN and LLS muscles converge at this point lateral to the nasal alar cartilage, where the nasolabial fold begins, providing a suitable point for injection. Approximately 0.2 to 0.3 mL per side was injected into the periosteum of the piriform fossa (upper part of the nasolabial fold, also known as the canine fossa), ensuring that there was no blood obstruction after negative aspiration of the facial artery after 20 s.

#### 2.2.2. Anterior Nasal Spine and Base of the Nose

Injection into the anterior nasal spine can decrease the activity of the nasal septal depressor muscle and is especially useful for central gingival smiles. This injection can also elevate the nasal tip. The two adjacent injections at the base of the nose are given to insert fibers between the LLSAN and nasal septum. Approximately 0.2 mL was injected over the anterior nasal spine at the bony level, and another 0.1 mL was injected in the retrotracer.

### 2.3. Measurements

The same operator took the color photographs from the front, with the subject posed in a natural head position, using a digital camera (Canon EOS 450 D, Madrid, Spain) [22]. All measurements were repeated twice to minimize measurement bias, with an interexaminer reproducibility of k = 0.975. Photographs were measured and analyzed using Image J software (National Institutes of Health, Bethesda, MD, USA) [23].

For each patient, measurements (mm) were taken from the lower edge of the upper lip to the incisal edge of 6 teeth: 13, 12, 11, 21, 22, and 23. The study focused on the evolution of the measurements over six time points or milestones, being before treatment (T0), immediately after treatment (T1), and five subsequent revisions: one week after treatment (T2), after one month (T3), and 3 months (T4), 6 months (T5), and 12 months (T6) after treatment.

**Figure 3:**
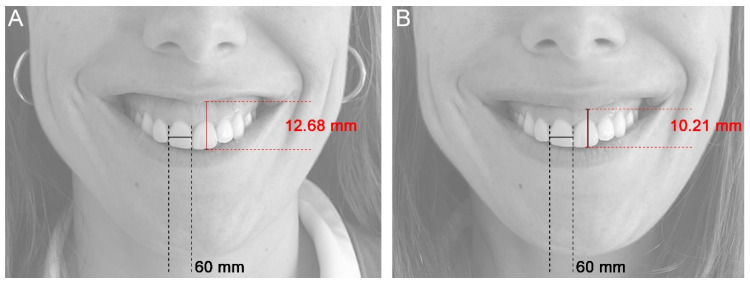
Control measurement of the central incisors to ensure that the pre- and post-treatment photographs were the same size and taken from the same angle. (**A**) Before treatment (T0), (**B**) immediately after treatment (T1).

A data matrix was configured and generated from the measurements, describing ten values for each patient and visit. These values corresponded to each of the measurement values of the original teeth (D13, D12, D11, D21, D22, and D23), the mean of central incisors 11 and 21 (G1), the mean of lateral incisors 12 and 22 (G2), the mean of canines 13 and 23 (G3), and the mean of the six original measurements taken (Mean D13 to D23). To analyze the evolution of the measurements over time and evaluate the efficacy of the technique, the difference in the measurements of the above parameters was evaluated: on the one hand, before treatment (T0) concerning all subsequent time points (Tx), and on the other hand, analyzing the difference after treatment (T1) and the five subsequent milestones. These data were expressed as a percentage improvement in reducing gingival exposure. To avoid variations in the size or angle of the photographs, a control measurement of the central incisors was taken as a constant parameter in the initial or control (T0) and subsequent (Tx) photographs (Figure 3).

### 2.4. Questionnaire

At the end of the 12 months of treatment, a satisfaction questionnaire after gingival smile treatment (Table 4), composed of 23 items, was carried out, which contemplated data regarding four domains: (1) patient satisfaction and confidence after the hyaluronic acid infiltration treatment of their gingival smile, (2) the level of pain, (3) the presence of bruising throughout all milestones of the process and in general terms, and (4) the recommendation of hyaluronic acid therapy, rated according to the degree of satisfaction from 1 to 4, according to a Likert scale.

### 2.5. Statistical Analysis

For this study of paired means (repeated in one group), and accepting an alpha risk of 0.05 and a beta risk of 0.2 in a bilateral contrast, 32 subjects must detect a difference equal to or more significant than 1 mm. A standard deviation of 2 mm is assumed.

Se ha estimado una tasa de pérdidas de seguimento del 0%. Data analysis was performed with SPSS v.11 statistical software for Windows. The descriptive statistical analysis (means, SD) was performed using the ten measurement values indicated, measured at the points above. The Kolmogorov–Smirnov test was used to determine the normal distribution of the data. For pairwise (or paired) comparison of the measurements between the different time points, a Student’s *t*-test was used when the distribution of the variables was normal and the Wilcoxon test when it was not, with a statistical significance of *p* < 0.05.

## 3. Results

Fifty subjects of both sexes who met the criteria for inclusion in the case series were included, counting 48 women and 2 men, ranging in age from 22 to 53 years (Table 5). Forty-one participants (forty women and one man) with a mean (SD) age of 33.8 ± 7.0 years completed the case series.

One participant did not attend the 1-week follow-up. All 50 cases attended the 1-month follow-up; 3 did not attend the 3-month follow-up; 7 did not attend the 6-month follow-up; and the final follow-up at 12 months was conducted on 41 patients. Table 6 shows the measurements taken throughout the time points evaluated.

**Figure 4:**
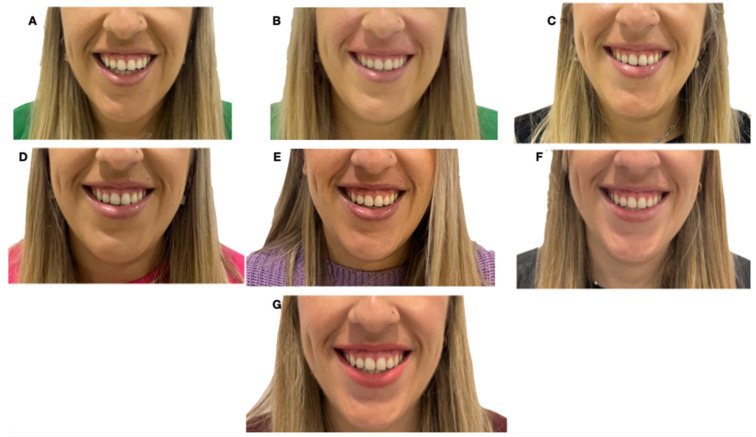
Frontal photographs of a patient with a smile before (**A**, T0) and after hyaluronic acid infiltration treatment (**B**, T1). (**C**–**G**) show successive follow-up visits: (**C**) T2, one week later, (**D**) T3, one month later, (**E**) T4, 3 months later, (**F**) T5, 6 months later, (**G**) T6, 12 months later.

All patients in the sample exhibited a decrease in the degree of gingival exposure. Figure 4 shows the GS improvement achieved for a patient at each time point with a reduction in gingival exposure before and after hyaluronic acid use. The average improvement after treatment (T1) was a 20.25% decrease in smile measurement. However, individually, the most significant reduction in the smile was achieved at one week (T2) after hyaluronic acid application in the D13 measurement, with an improvement of 21.77%.

**Figure 5:**
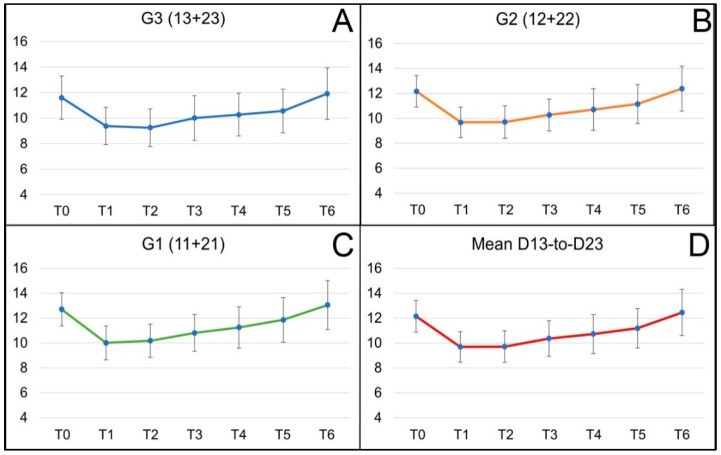
Evolution of the means (and SD) of the GS measurements for groups’ G3 canines (**A**), G2 lateral incisors (**B**), G1 central incisors, (**C**) and the mean of the six tooth measurements, Mean D13 to D23 (**D**), in successive visits. Milestones or time points are defined as T0, before treatment; T1, immediately after treatment; T2, 1 week after treatment; T3, 1 month; T4, 3 months; T5, 6 months; and T6, 12 months after treatment.

The mean baseline pretreatment score for the mean of the six teeth (Mean D13 to D23) of the distance from the upper lip edge to the incisal edge of the teeth was 12.15 mm, with a standard deviation of 1.27 mm. Immediately after treatment, a remarkable statistically significant decrease in this distance was observed, down to 9.69 mm (SD 1.23 mm) (*p* < 0.001). For this group, a gradual increase in measurements followed in successive revisions, although the values continued to show significant differences (*p* < 0.001). Levels close to the initial ones were only reached after one year, where the values compared were statistically equal (Figure 5D).

**Figure 6:**
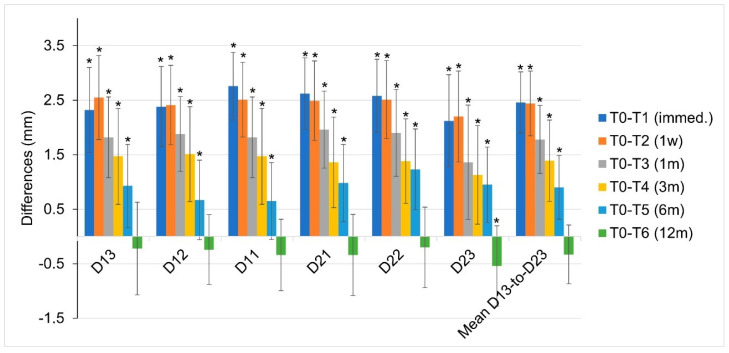
Differences (mm) between the measurement before and after treatment at T0–T1 (immed.) and with respect to the rest of the time points for each of the teeth evaluated and the mean of the six tooth measurements, Mean D13 to D23. Taking the value 0 mm as the measurement before treatment (T0), the asterisks indicate a statistically significant difference between the measurements before treatment and those taken at each time point (T1, T2, T3, T4, T5 and T6).

This trend of substantial primary improvement and regression to the original state is also observed when comparing the differences between the data of individual teeth (D13, D12, D11, D21, D22, D23) before treatment (T0) with those immediately after treatment (T1) and the rest of the time points before one year, i.e., up to and including the 6-month checkup. A statistically significant difference is observed (*p* < 0.001). The maximum difference is reached at measurement D11, with values up to 2.76 ± 1.24 mm difference before and just after infiltration (Figure 6). However, at the 12-month visit, there are no significant differences between the measurements taken before and those at that visit, except for tooth 23, where the GS is more important after 12 months than at the start of treatment. Despite the evident trend in the evolution of the differences, a specific variability is indicated by the error bars at each measurement point.

When comparisons are made between time point T1 (after treatment), once the maximum decrease in GS has been achieved, and the successive time points, the results show that there are no differences between the measurements after treatment (T1) and the following week (T2) (*p* > 0.05) in any of the measurements and variables analyzed, either in teeth (Dx) or groups (Gx) or in the mean of all teeth (Mean D13 to D23) (blue bars, Figure 6), even though, in canines, the reduction in the gingival smile shows a mean clinical improvement one week after infiltration. Statistical differences start to appear from the one-month post-treatment visit (T3) (*p* < 0.001 for all measurements), in which a gradual increase in measurements follows in successive revisions.

From the present data, it can be deduced that, in general terms, 20% of the original gingival exposure was recovered at one month, followed by an additional 40% recovery at three months. At six months follow-up, a 40% gain in gingival smile reduction was maintained with a gradual return to the initial state, reaching full recovery at 12 months.

**Figure 7:**
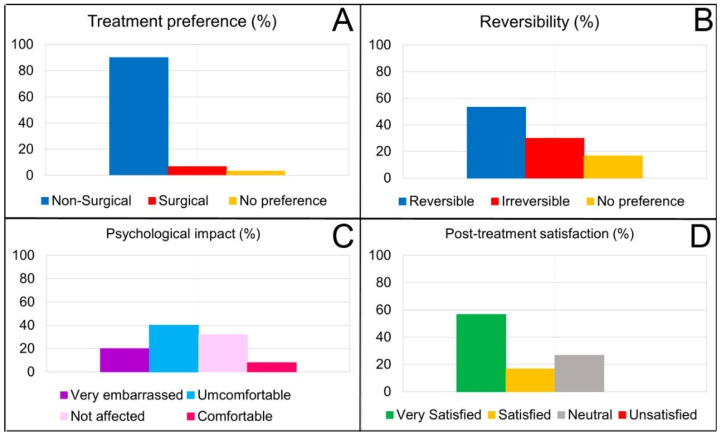
Results in percentages of the preference for surgical treatment or not (**A**) and reversible or not (**B**) for the correction of the gummy smile (n = 50). Percentages of the psychological impact or affectation of GS on treated patients (**C**); proportion of satisfaction among patients after treatment (**D**) (n = 41).

In the Gingival Smile Treatment Selection Survey conducted during the first visit, patients were asked about their therapeutic preferences for treating their GS. Almost 70% of them had consulted an oral health professional to evaluate their GS case, and 90% indicated a preference for nonsurgical treatment to correct their gingival smile (Figure 7A). Similarly, 53.3% preferred their treatment as reversible therapy, and 17% did not care (Figure 7B).

Regarding the psychological impact of having GS (Gingival Smile Psychological Impact Survey), 64% of the patients treated had experienced embarrassment or low self-esteem due to their gingival smile. In addition, 40% indicated that they felt uncomfortable with their smile, and up to 20% of the participants said they were very embarrassed (Figure 7C).

**Figure 8:**
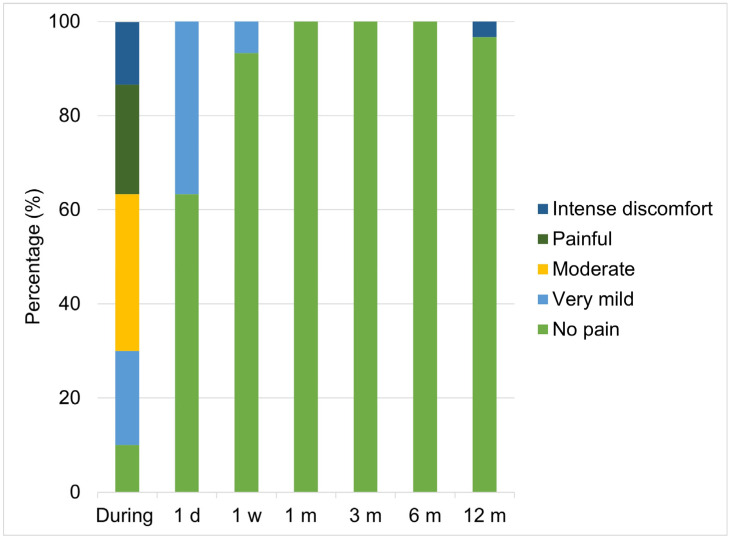
Results in percentages of the level of pain reported by the patients in the study throughout the entire process. Abbreviations: d, day; w, week; m, month.

At the last patient follow-up visit (T6, 12 months), a satisfaction questionnaire about the hyaluronic acid infiltration treatment was carried out, which included 23 items. From the data obtained (n = 41), none of the patients experienced significant side effects or complications after treatment, with only 13% reporting mild effects and 97% reporting no complications (Figure 8). Improvements in personal confidence following hyaluronic acid infiltration treatment were reported for 60% of participants. In addition, up to 82.1% indicated that they were satisfied or delighted after the proposed treatment (Figure 7D), and 100% of the sample would recommend it.

## 4. Discussion

This study evaluated the efficacy of hyaluronic acid as a myomodulator in treating gingival smiles, observing significant changes in smile measurements at various post-treatment stages. The results indicate a remarkable initial reduction in gingival exposure, followed by a gradual recovery toward the initial values, suggesting a progressive adaptation and adjustment of the facial muscle. Therefore, the null hypothesis is partially rejected. Caution should be exercised about measurements taken on smiles captured by photography. Repairing a natural smile in several sessions is a complex objective, and photographic capture, although it facilitates measurement, is not always the best technique. The predominance of women in our study may be explained by the higher prevalence of gingival smiles in women, as mentioned in the introduction. Previous studies have indicated that OS affects between 10.5% and 29% of young adults, with approximately 14% of women being affected and 7% of men [1]. This difference in prevalence is likely reflected in our sample, given that women tend to be more concerned with aesthetic issues and may have a greater tendency to seek corrective treatments for the gingival smile [2,3]. This female predominance, therefore, is consistent with existing epidemiological data on the condition. Analyzing the anatomical elements is fundamental to understanding and adequately treating GS [24]. Thus, the height of the alveolar bone determines the proportion of the gingiva visible when smiling, as well as the mobility of the upper lip [13,15]. The length and position of the teeth affect the exposure of the gingiva depending on how the teeth align in the arch. An altered passive eruption may increase gingival visibility when smiling because of less lip coverage [25]. Excessive vertical growth of the maxilla results in facial disproportion that may expose more gingivae [26]. The hyperactivity of the upper lip muscles leads to excessive lifting of that lip during smiling, showing more gingiva [24,27,28]. These anatomic and functional factors contributing to GS provide a framework for evaluating and treating this condition in affected patients [27]. However, there is currently no consensus on a therapeutic approach to GS that provides predictable outcomes, regardless of its etiology [6].

Several therapeutic methods for correcting gummy smiles have been proposed, including invasive, non-invasive, and minimally invasive approaches [1,2,6,7,10,11,21,24,26,29]. Nonetheless, invasive procedures have been associated with high morbidity [26,30]. Therefore, less invasive therapeutics that reduce risks and recovery time with evident effectiveness rates are an attractive alternative for patients and clinicians.

The fact that we incorporated patients without discriminating between different gummy smile patterns may detract from the study’s validity, since different types of gummy smiles are tributary for different kinds of treatments. The data from the present study show a trend in the change in the distance from the upper lip edge to the incisal edge over time after treatment, as the resulting compression of the hyaluronic acid appears to interfere with muscle contraction, and the vertical action of LLSAN and nasal septum [1]. According to the results, when specifically analyzing each tooth, it is noted that the front teeth, such as the central incisors (11 and 21), tend to show faster and more significant recovery in mean measurements compared to both lateral teeth (12 and 22) and canines (13, 23) (Figure 4). This could be due to structural differences or the different functional loads each tooth type handles [31]. In addition, lateral teeth exhibit more significant variability, especially in the late follow-up stages, which could indicate differential adaptation to treatment-induced changes [32].

Regarding variability measured by standard deviation, relative consistency was observed over time, with a slight increase during the 3- and 6-month revisions. This increase could indicate variability in individual responses as the muscles stabilize or adjust to the treatment received [12], especially when considering the possible difference in the etiology of GS [11,24,28]. For the 1-year review, the standard deviation reached its highest values, which may reflect more marked differences in how each individual responds to long-term treatment [29].

A phenomenon observed in the T6 measurement, which showed an increase in gingival exposure when smiling after one year, may be explained by the fact that patients become more aware of their smiles after the various treatments and photographic follow-ups. This may lead to them smiling more freely and naturally during the photographic sessions compared to the beginning of the study, which influences the measurements by reflecting a wider smile width. This greater awareness and naturalness may have influenced the differences observed at T6.

Overall, the trajectory of the measurements and their variabilities illustrates a dynamic process of muscle adjustment and post-treatment adaptation. Fillers can be injected superficially into the muscle fibers, which can induce a partial decrease in muscle function, which helps treat the gummy smile. They can also be injected deeply, thus compressing the muscle fibers [1,28]. Although there is a general trend toward recovery of the original measurements, the speed and magnitude of this recovery vary, probably influenced by the location of the product [1,33] and individual characteristics of response to treatment [9]. These observations may provide valuable data on the efficacy and long-term effects of hyaluronic acid procedures as myomodulators [9].

After treatment with hyaluronic acid infiltration, an average decrease of 2.5 mm in gingival exposure was observed during smiling. During the week after treatment, a constant reduction was maintained. At one month, 20% of the original gingival exposure was restored, followed by an additional 40% restoration at three months. A 40% gain in gingival smile reduction was maintained at six months follow-up, reaching full recovery at 12 months post-treatment. These data improve expectations to those provided by research using botulinum toxin, in which the effect lasts only 3 to 6 months, depending on the brand of BT [19,34]. In addition, botulinum toxin injection, despite being a simple and safe procedure, can be associated with ptosis or lengthening of the upper lip and asymmetry of the smile, with consequent unsightly effects [6,35] and cannot be removed in case of unpleasant results. Injectable fillers, such as hyaluronic acid infiltration, have a unique and vital role, as they can support weak muscle action and locally block overactive muscles [9].

While a 20% reduction in gingival display after treatment may seem modest, we believe it is clinically relevant in the context of the aesthetic improvement perceived by patients. In addition, it is important to note that this type of treatment attracts patients who have sought non-surgical alternatives due to the limitations and risks of invasive procedures. Here, 90% of the patients surveyed preferred a non-surgical and reversible treatment, and 82.1% were satisfied or very satisfied with the results. Although the initial reduction of 20% is remarkable, the positive perception of the patients and the improvement in their confidence after treatment (reported by 60% of the participants) reinforce the importance of these results from a clinical and psychological perspective. We believe that these results are significant for the objective reduction in millimeters and the positive impact on the patient’s self-esteem and aesthetic perception. Since the treatment proposal of Diaspro et al. [1], we can conclude that this new hyaluronic acid infiltration technique proved effective in the treatment of gingival smiles in the patients evaluated in our study. The precise application of small amounts of hyaluronic acid at strategic points significantly reduced gingival exposure during smiling. Moreover, the results were maintained for up to 6 months with a 40% gain in gingival smile reduction except for the duration indicated by the manufacturer, which is 8 to 12 months. Therefore, we conclude that proper product selection is essential to achieve good, longer-lasting results. As pointed out by Diaspro et al. [1] in their study, it is confirmed that the product must have an elastic modulus G’ capable of tolerating the dynamic forces that occur in smile movement.

The literature describes the gummy smile as an aesthetic problem affecting people’s quality of life, as it affects their self-esteem and social perception [5,6,36], among other factors, due to smile-avoidance behavior. Even in certain cultures, the smile has been identified as the most important facial aesthetic feature [37]. The present study explored several dimensions of the gingival smile, including the psychological impact on patients, an aesthetic aspect that significantly concerns them. In the pretreatment surveys, the majority (89.7%) expressed a predominant concern about excessive gingival exposure, which agrees with the existing literature highlighting how gingival exposure is considered an essential aesthetic deviation for many individuals [5,31]. Our data also revealed that more than half of the respondents preferred a reversible approach to the correction of their GS and expressed an overwhelming preference (90%) for nonsurgical treatments, reflecting trends observed in previous studies [38] indicating a bias toward less invasive methods due to the benefits of lower risk and faster recovery [10].

The data provided on patient satisfaction should be interpreted with caution, as the surveys used have not been previously validated to ensure their applicability and validity. Nevertheless, in our research, the treatment of the gingival smile with hyaluronic acid infiltrations crucially influenced GS’s psychological impact on treated patients, where 60% of participants reported improvements in their confidence after treatment. This finding is consistent with the literature suggesting that aesthetic improvements in the smile contribute significantly to individuals’ self-esteem and social perception [5,36,37]. The high post-treatment satisfaction and recommendation rates (82.1% and 100%, respectively) observed in our study highlight the effectiveness and acceptability of the treatments employed, which is crucial for future clinical recommendations and alignment with patients’ expectations [6]. These high satisfaction values are supported, among other issues, by the fact that there were practically no side effects during the process and especially throughout the entire follow-up period for this technique, both in terms of the quantification of pain and in terms of hematomas and inflammation (Figure 7).

These results support the continued need for innovation and improvement in treatment options for the gummy smile, emphasizing the importance of considering both aesthetic outcomes and psychological impacts on patients.

Although the average reduction of 2 mm in gingival exposure may seem modest, it is important to note that these results were achieved using only 1 mL of hyaluronic acid. This leads us to consider that, with a larger volume of product, the results in terms of millimeter reduction could be even more significant and potentially increase the duration of the effect. As mentioned above, the initial 20–25% improvement (about 2.5 mm) was maintained at a 40% reduction at six months before gradually returning to the original values at 12 months. However, we believe that this procedure is promising because it offers a minimally invasive and reversible option, especially for patients seeking to avoid surgery. It is crucial to determine whether using higher doses of hyaluronic acid could increase the millimeter reduction of the gingival smile and prolong the duration of the clinical effect. Furthermore, as indicated, the psychological impact of the gummy smile is significant, with 60% of participants reporting improved confidence after treatment and more than 80% of patients reporting satisfaction with the results, suggesting that even a 2 mm improvement can have a visible and appreciated impact. However, exploring higher doses may be a key area for future studies to maximize the benefits.

## 5. Conclusions

This study corroborated the efficacy of this new hyaluronic acid infiltration technique in the correction of the gingival smile, showing an immediate average reduction of 2.5 mm in gingival exposure and maintaining 40% of this improvement at six months. The acceptance of the treatment was remarkably high, with more than 80% of patients expressing significant satisfaction and a solid propensity to recommend the procedure. The preference for non-surgical solutions and the positive perception of the results, which included improvements in personal confidence and facial aesthetics, underscore the viability of hyaluronic acid as a less invasive alternative with long-lasting effects and minimal side effects. These findings encourage continued research to refine techniques and extend the durability of the impact, providing patients with affordable, low-impact aesthetic treatments to improve their quality of life.

## Figures and Tables

**Table 1:** Inclusion and exclusion criteria for the study (mental disorders were screened by a negative response to a visit to a psychologist/psychiatrist or absence of taking medication for this type of disorder).

Inclusion Criteria	Exclusion Criteria
Patients between 18 and 55 years of age.	History of neuromuscular disorder (e.g., myasthenia gravis, Eaton–Lambert syndrome).
Exposure of the entire dental crown/teeth and a contiguous gingival band > 3 mm.	Orthodontic or crown-based treatments initiated in the anterior sector during the study period.
No history of previous treatment for gummy smile correction, with hyaluronic acid or botulinum toxin being the area of interest, within the last year.	Patients who have previously undergone any gummy smile treatment.
Patients who want an aesthetic correction of the smile without surgery.	Gingival smile involving hyperactivity of the zygomatic muscles
Commitment is needed to follow the study follow-up protocol and complete the questionnaires.	Patients with underlying mental disorders, psychological instability, or who had questionable motives and unrealistic expectations were not included.

**Table 2:** Gingival Smile Treatment Selection Survey.

Questions	Categories
1. What is your main concern regarding your gingival smile?	(a) Excessive gum exposure
	(b) Asymmetry of the gingival contour
	(c) Other concerns
2. Do you prefer surgical or non-surgical treatment to correct your gummy smile?	(a) Surgical
	(b) Nonsurgical
	(c) No preference
3. How much do you value the duration of treatment?	(a) Rapid results, even if temporary
	(b) Long-lasting results, even if the treatment takes more time
	(c) No preference
4. Do you have any health conditions or allergies that may affect the treatment choice?	(a) Yes
	(b) No
	(c) Not sure
5. How willing are you to undergo a surgical procedure?	(a) Fully willing
	(b) Somewhat reluctant, but would consider the surgical option.
	(c) Unwilling
6. How significant is the reversibility of the treatment to you?	(a) I don’t care if the treatment is reversible or irreversible.
	(b) I prefer reversible treatment
	(c) I prefer an irreversible treatment if it is more effective.
7. Have you consulted an oral health professional to evaluate your case?	(a) Yes, I have already consulted a professional.
	(b) No, I have not yet consulted a professional.
	(c) No, but I plan to do so soon
Preferred: Periodontal Coronary Lengthening Treatment	☐ Yes | No ☐
Preference: Botulinum Toxin Gummy Smile Treatment	☐ Yes | No ☐
Preference: Orthognathic Surgery	☐ Yes | No ☐
Preference: Lip replacement surgery	☐ Yes | No ☐
Preference: Treatment of the gummy smile with hyaluronic acid infiltration	☐ Yes | No ☐
Preference: I have no specific preference	☐ Yes | No ☐

**Table 3:** Gingival Smile Psychological Impact Survey.

Questions	Categories
1. How do you feel about your gummy smile?	(a) I feel very insecure and embarrassed about my smile.
	(b) I feel uncomfortable, but try to accept my smile as it is.
	(c) I am not emotionally affected by my gummy smile.
	(d) I am proud of my gummy smile.
2. Have you ever experienced embarrassment or low self-esteem because of your gummy smile?	(a) Yes, frequently.
	(b) Yes, occasionally.
	(c) No, never.
3. How do you think your gummy smile has affected your self-confidence?	(a) My self-confidence has decreased significantly.
	(b) It has impacted my confidence, but not significantly.
	(c) It has not affected my confidence at all.
4. Have you ever avoided smiling or laughing openly because of your gummy smile?	(a) Yes, I avoid smiling and laughing openly all the time.
	(b) Yes, I avoid it in some specific social situations.
	(c) I do not avoid smiling or laughing openly because of my gummy smile.
5. Have you ever received negative comments or teasing because of your gummy smile?	(a) Yes, frequently.
	(b) Yes, occasionally.
	(c) No, never.
6. Have you felt motivated to seek a solution to correct your gummy smile because of its psychological impact?	(a) Yes.
	(b) Yes, but I have not yet taken any action.
	(c) No, I do not need to correct my gummy smile.
7. Have you sought psychological support or counseling to deal with the emotional effects of your gummy smile?	(a) Yes, I have sought professional support and advice.
	(b) No, but I have considered seeking support in the future.
	(c) No, I have not sought support or counseling.

**Table 4:** Summary of the questions of the satisfaction survey after gummy smile treatment. Level of pain and bruising.

*Questions* Categories			
*1. How do you feel after treating your gummy smile?* Very Satisfied ☐ 1 ☐ 2 ☐ 3 ☐ 4 Dissatisfied			
*2. How has your level of self-confidence changed after treatment?* Significant increase ☐ 1 ☐ 2 ☐ 3 ☐ 4 I am no longer sure			
*3. How has treatment affected your social life and interactions with others?* Improved ☐ 1 ☐ 2 ☐ 3 ☐ 4 No improvement			
*4. What is your level of satisfaction with the aesthetic results of the treatment?* Very Satisfied ☐ 1 ☐ 2 ☐ 3 ☐ 4 Dissatisfied			
*5. Would you recommend the gingival smile correction treatment to your family and friends?* Yes ☐ 1 ☐ 2 ☐ 3 ☐ 4 No			
*6. Have you experienced any side effects or complications after treatment?* ☐ No ☐ Yes, but mild ☐ Yes, completely			

*Overall satisfaction with treatment results* Very Satisfied ☐ 1 ☐ 2 ☐ 3 ☐ 4 ☐ 5 Dissatisfied			
Treatment *recommendation* ☐ Yes ☐ No			
**Pain level**			
In the treatment	No discomfort ☐ 1 ☐ 2 ☐ 3 ☐ 4 ☐ 5 Severe discomfort		
One day later	No discomfort ☐ 1 ☐ 2 ☐ 3 ☐ 4 ☐ 5 Severe discomfort		
One week later	No discomfort ☐ 1 ☐ 2 ☐ 3 ☐ 4 ☐ 5 Severe discomfort		
One month later.	No discomfort ☐ 1 ☐ 2 ☐ 3 ☐ 4 ☐ 5 Severe discomfort		
Three months later	No discomfort ☐ 1 ☐ 2 ☐ 3 ☐ 4 ☐ 5 Severe discomfort		
Six months later	No discomfort ☐ 1 ☐ 2 ☐ 3 ☐ 4 ☐ 5 Severe discomfort		
12 months later	No discomfort ☐ 1 ☐ 2 ☐ 3 ☐ 4 ☐ 5 Severe discomfort		
**Presence of bruising and swelling**			
No bruising and swelling ☐ 1 ☐ 2 ☐ 3 ☐ 4 ☐ 5 Marked			
In the treatment	☐ Yes ☐ No	1 day later	☐ Yes | No ☐ No
One week later.	☐ Yes ☐ No	One month later	☐ Yes | No ☐ No
3 months later	☐ Yes | No ☐ No	Six months later	☐ Yes ☐ No
12 months later	☐ Yes | No ☐ No		

**Table 5:** Demographic characteristics (age, sex, and others) of patients included in the study (n = 41).

Variable	n (%)	Variable	n (%)
*Age (years)*		*Sex*	
18–24	4 (9.8)	Male	1 (2.4)
25–34	20 (48.7)	Female	40 (97.6)
35–44	12 (29.3)		
45–55	5 (12.2)	*Ethnicity*	
Mean ± SD	33.8 ± 7.0	White	37 (90.2)
		Hispanic/Latino	4 (9.2)
*Smoking status*			
Non-Smoker	35 (85.4)	*Allergies*	
Smoker	6 (14.6)	Yes	2 (4.9)
Cigarettes/day in smokers (Mean ± SD)	7.8 ± 5.2	No	39 (95.1)

**Table 6:** Descriptive statistics of measurements (mm) over time.

Variable (Measurements)	TIME, Mean (SD)						
	T0 (Baseline)	T1 (immed.)	T2 (1 w)	T3 (1 m)	T4 (3 m)	T5 (6 m)	T6 (12 m)
D13	11.76 (1.70)	9.44 (1.79)	9.20 (1.68)	9.94 (1.88)	10.26 (1.65)	10.77 (1.78)	11.90 (2.06)
D12	12.20 (1.29)	9.82 (1.34)	9.76 (1.41)	10.32 (1.38)	10.68 (1.66)	11.47 (1.55)	12.42 (1.83)
D11	12.84 (1.38)	10.08 (1.46)	10.31 (1.56)	11.02 (1.52)	11.34 (1.74)	12.19 (1.86)	13.20 (1.99)
D21	12.56 (1.39)	9.94 (1.38)	10.06 (1.28)	10.60 (1.53)	11.15 (1.68)	11.54 (1.86)	12.90 (2.05)
D22	12.12 (1.48)	9.54 (1.33)	9.65 (1.39)	10.22 (1.48)	10.72 (1.90)	10.84 (1.75)	12.32 (1.98)
D23	11.44 (2.02)	9.32 (1.46)	9.29 (1.54)	10.08 (1.83)	10.28 (2.01)	10.35 (1.89)	11.93 (2.22)
G3 (13+23)	11.60 (1.69)	9.38 (1.46)	9.25 (1.47)	10.01 (1.75)	10.27 (1.67)	10.56 (1.71)	11.92 (2.02)
G2 (12+22)	12.16 (1.26)	9.68 (1.22)	9.70 (1.30)	10.27 (1.28)	10.70 (1.67)	11.15 (1.56)	12.38 (1.80)
G1 (11+21)	12.70 (1.33)	10.01 (1.36)	10.18 (1.33)	10.81 (1.48)	11.25 (1.66)	11.86 (1.80)	13.05 (1.97)
Mean D13-to-D23	12.15 (1.27)	9.69 (1.23)	9.71 (1.27)	10.36 (1.43)	10.73 (1.57)	11.19 (1.58)	12.45 (1.85)

Abbreviations: SD: standard deviation; immed.: immediately after treatment; w: week; m: months.

## Data Availability

The data presented in this study are available on request from the corresponding author.
